# Integrating Imaging and Nutrition: Chest CT Muscle Analysis in Adults with Cystic Fibrosis

**DOI:** 10.3390/nu17182940

**Published:** 2025-09-12

**Authors:** Virginia Soria-Utrilla, Ana Piñar-Gutiérrez, Francisco José Sánchez-Torralvo, Antonio Adarve-Castro, Nuria Porras, Andrés Jiménez-Sánchez, María Esther Quintana-Gallego, Casilda Olveira, María Victoria Girón, Gabriel Olveira, Pedro Pablo García-Luna

**Affiliations:** 1 Endocrinology and Nutrition Department, Hospital Regional Universitario de Málaga, 29010 Málaga, Spain; virginiasoriau@uma.es (V.S.-U.); fransancheztorralvo@gmail.com (F.J.S.-T.); nporrasperez@hotmail.com (N.P.); 2 Department of Medicine and Dermatology, Faculty of Medicine, University of Malaga, 29016 Málaga, Spain; casi1547@separ.es; 3 Endocrinology and Nutrition Department, Hospital Universitario Virgen del Rocío, 41013 Sevilla, Spainandres.jimenez.sanchez.sspa@juntadeandalucia.es (A.J.-S.); garcialunapp@yahoo.es (P.P.G.-L.); 4 Instituto de Investigación Biomédica de Málaga (IBIMA)-BIONAND Platform, 29590 Málaga, Spain; 5 Radiology Department, Hospital Universitario Virgen de la Victoria, 29010 Málaga, Spain; elaadarve@hotmail.com; 6 Instituto de Biomedicina de Sevilla (IBiS), Hospital Universitario Virgen del Rocío/CSIC/Universidad de Sevilla, 41013 Sevilla, Spain; mariae.quintana.sspa@juntadeandalucia.es; 7 Pneumology Department, Hospital Universitario Virgen del Rocío, 41013 Sevilla, Spain; 8 Pneumology Department, Hospital Regional Universitario de Málaga, 29010 Málaga, Spain; consultaenfermerafq.bq@gmail.com; 9 Biomedical Research Networking Center in Diabetes and Associated Metabolic Disorders (CIBERDEM), Instituto de Salud Carlos III, 29010 Málaga, Spain

**Keywords:** body composition, cystic fibrosis, computed tomography, malnutrition, muscle mass, nutritional assessment

## Abstract

**Background:** Computed Tomography (CT) is considered a highly accurate tool for assessing body composition. The aim of this study is to assess the usefulness of chest CT for malnutrition diagnosis in people with cystic fibrosis (PwCF), compared with other body composition techniques, as well as to assess possible associations with nutritional and respiratory status. **Methods**: A cross-sectional study was carried out in clinically stable adult PwCF. Subjects who had undergone a CT including the twelfth thoracic vertebra (T12) during the 6 months prior to or after our assessment were included and body composition was assessed using FocusedON-BC. The results were compared with anthropometry, bioelectrical impedance analysis (BIA), muscle ultrasonography, and handgrip strength (HGS). Respiratory parameters were collected, and nutritional status was assessed using Global Leadership Initiative on Malnutrition (GLIM) criteria. **Results**: A total of 55 PwCF were included. Muscle area assessed by CT correlated significantly with fat-free mass determined by BIA (*r* = 0.725) and anthropometry (*r* = 0.645), muscle mass evaluated by ultrasonography (*r* = 0.657), HGS (*r* = 0.593), Bhalla score (*r* = 0.403), and FEV1 (*r* = 0.488). Differences were observed when comparing muscle area in CT based on the Bhalla score (94.6 ± 21.1 cm^2^ in normal/mild involvement vs. 79.3 ± 20.9 cm^2^ in moderate/severe involvement; *p* = 0.009) and on nutritional status (96.3 ± 17.9 cm^2^ in normo-nourished vs. 75.9 ± 22.1 cm^2^ in malnourished; *p* < 0.001). **Conclusions**: In adult PwCF, measurements obtained from CT image analysis correlate adequately with anthropometry, BIA, muscle ultrasound, and HGS. Muscle area in CT is related to nutritional and respiratory status.

## 1. Introduction

Cystic fibrosis (CF) is an autosomal recessive genetic disease caused by mutations in the CFTR gene, resulting in impaired ion transport in different organs and tissues, including respiratory, hepatobiliary, gastrointestinal, and reproductive systems, as well as pancreas and sweat glands, making CF a multisystem disease [1].

Additionally, CF has historically been associated with malnutrition and progressive deterioration in nutritional status [2]. Currently, despite interventions focused on its prevention, malnutrition rates are close to 25% [3,4], reaching up to 40–50% depending on the tool used [5,6]. Malnutrition affects respiratory muscles and, thus, lung function [7], being a predictive risk factor for morbimortality in people with CF (PwCF) [2,8]. Hence, there is a need for malnutrition assessment and treatment in PwCF [5,9].

Classically, malnutrition has been defined by body mass index (BMI) < 18.5 kg/m^2^ for adults with CF. However, current guidelines recommend longitudinal assessment of body composition for a comprehensive nutritional status evaluation, since fat mass and fat-free mass (FFM) correlate better with respiratory status. In fact, increased adiposity along with low FFM may be even more detrimental to pulmonary function than a high BMI [9]. Additionally, it is also possible to be obese or overweight and present FFM depletion [10].

Anthropometrical measurements, bioelectrical impedance analysis (BIA), and dual-energy X-ray absorptiometry (DXA) have been validated in CF [2,6]. In a previous study, we observed that muscle ultrasonography of quadriceps rectus femoris correlate adequately with the other body composition techniques [2]. Meanwhile, handgrip strength can be more sensitive to changes in muscle function, making a good marker of renutrition [9].

However, there is no single parameter that accurately reflects nutritional status. Therefore, those body composition measurements and techniques that are practical, sensitive, and specific, while aligning with other tools, are of significant importance [11]. Among these techniques, Computed Tomography (CT) is considered a highly accurate tool for assessing body composition. Although its routine use for this purpose is not feasible, due to the radiation dose involved, images acquired for other medical purposes can be used to assess body composition [12,13]. Currently, muscle mass measured at the level of the third lumbar vertebra (L3) is considered the reference standard for estimating body skeletal muscle mass by CT. However, chest CT scans do not always include L3 [14]. Some authors have suggested that the use of CT at the level of the twelfth thoracic vertebra (T12) is a valid tool to assess body composition in PwCF [13].

In line with the above, our hypothesis is that CT could be an appropriate technique for measuring muscle mass in the nutritional assessment of PwCF, and that it would have good agreement with other diagnostic tools.

Thus, the aim of this study is to assess the usefulness of CT for malnutrition diagnosis in PwCF, comparing the results with other body composition techniques such as anthropometry, BIA, handgrip strength, and nutritional ultrasound, as well as to determine its relationship with respiratory and nutritional status.

## 2. Materials and Methods

### 2.1. Ethics

This study was approved by the Research Ethics Committee of Malaga on 30 March 2021 (reference number #30032021). All subjects received a full explanation of the study objectives and procedures, and signed the informed consent form before participating. The study was conducted in accordance with the Declaration of Helsinki.

### 2.2. Study Design and Participants

**Figure 1:**
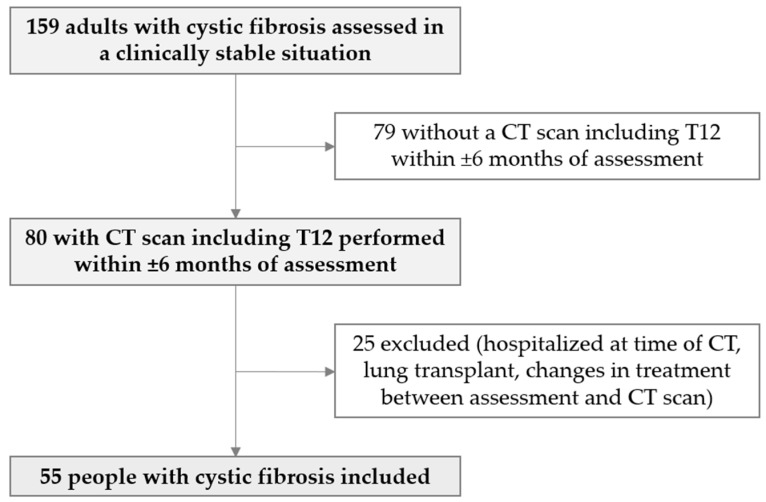
Study flow diagram.

This was a cross-sectional observational study of routine clinical practice, conducted at two centers: the Hospital Regional Universitario de Malaga and the Hospital Universitario Virgen del Rocío. Adult PwCF in a clinically stable situation assessed at the Nutrition Unit were selected. We included subjects who had undergone a CT scan that included T12 within the 6 months before or after our assessment. Exclusion criteria included hospitalization or respiratory exacerbation at the time of CT performance, lung transplantation, or treatment modification, especially initiation of ETI, between our assessment and the CT scan, so 55 PwCF were finally included (Figure 1).

### 2.3. Morphofunctional Assessment

Nutritional assessment included the following:

- Height, weight, and BMI.
- Skinfold thickness of triceps, biceps, subscapularis, and supra-iliac regions using constant-pressure calipers Holtain (Holtain Limited, Crymych, UK) or Harpenden (Realmet Institute, Vic, Spain). Fat mass and FFM were estimated according to the formulas of Siri and Durnin [15,16].
- BIA using TANITA MC980MA (TANITA Corporation, Tokyo, Japan) to assess total body composition, including raw phase angle, fat mass, and FFM. FFM index (FFMI) was calculated for anthropometry and BIA. Low FFMI was defined according to Global Leadership Initiative on Malnutrition (GLIM) consensus [17].
- Handgrip strength measurement using a Jamar dynamometer (Asimow Engineering Co., Los Angeles, CA, USA).
- For patients assessed at the Hospital Regional Universitario de Malaga, muscle ultrasonography of rectus femoris muscle using a 10–12 MHz probe and an Esaote MyLab Gamma device (Esaote, Genova, Italy). Measurements were acquired at point one third of the distance along a line drawn from the upper edge of the patella to the anterosuperior iliac spine, without applying pressure with the probe. Parameters measured included *X*-axis (major transversal axis of rectus anterior), *Y*-axis (minor anteroposterior axis of rectus anterior), muscular area of rectus anterior (MARA), and transverse subcutaneous adipose tissue (TSAT) [18]. MARA index (MARAI) was calculated.

### 2.4. Computed Tomography (CT) Image Analysis

Body composition was assessed using CT images acquired as part of routine clinical practice.

CT image processing was performed using FocusedON-BC software from ARTIS Development (version 1.0). This software incorporates an artificial intelligence-assisted semiautomatic labeling tool able to perform body mass segmentation automatically. Hounsfield Unit (HU) thresholds used for segmentation are as follows: −29 to +150 for muscle mass, −190 to −30 for subcutaneous adipose tissue (SAT) and intramuscular adipose tissue (IMAT), and −150 to −50 for visceral adipose tissue (VAT). Cross-sectional areas (cm^2^) for each tissue type are automatically calculated based on the pixel size and the number of labeled pixels for each tissue.

Once CT images were uploaded, we selected the CT slice at the midpoint level of T12 and processed it, with manual adjustments if needed. Thus, since the primary function of FocusedON-BC is to accelerate the image analysis by providing an initial segmentation that the researcher can then validate and, if necessary, correct, human judgment prevails as the final authority, eliminating any potential bias that the model may have introduced.

We obtained the area, percentage, and mean HU of both muscle and adipose tissue, including IMAT, SAT, and VAT. Skeletal muscle mass index (SMI) was calculated from muscle area and height.

**Figure 2:**
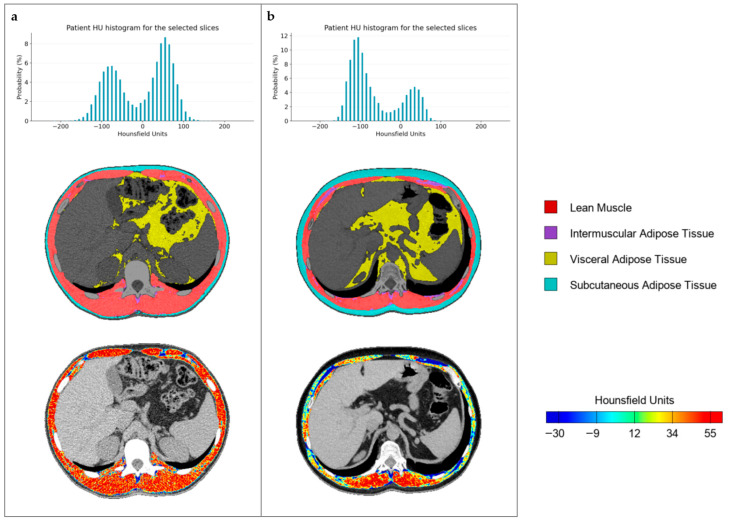
Graphical information extracted from FocusedON-BC: (**a**) patient with normal BMI and normal muscle mass; (**b**) patient with normal BMI and low muscle mass. Both patients have a similar BMI.

Additionally, FocusedON-BC provides graphical information that allows a quick qualitative overview of the results (Figure 2).

### 2.5. Assessment of Nutritional Status

Malnutrition was defined according to GLIM criteria, for which the presence of at least one phenotypic criterion and one etiologic criterion was required. All patients were found to meet at least one etiological criterion, as CF is considered a chronic inflammatory condition. Regarding phenotypic criteria, patients were identified based on unintentional weight loss higher than 5% in 6 months, BMI below 20 kg/m^2^, and decreased FFMI in BIA [17].

### 2.6. Assessment of Respiratory Status

Respiratory exacerbations occurring within the year prior to the evaluation were classified as mild/moderate or severe (if required hospitalization and/or intravenous antibiotics).

Spirometry was performed using a JAEGER pneumotachograph (Jaeger Oxycon Pro^®^, Erich Jaeger, Würzberg, Germany) to measure forced expiratory volume in 1 s (FEV1), forced vital capacity (FVC), and FEV1/FVC ratio [19].

Initial microorganism colonization was determined by the presence of at least three positive sputum cultures, regardless of their persistence at the time of this study.

Pulmonary radiological severity was evaluated by a radiologist with expertise in thoracic radiology using Bhalla score [20].

### 2.7. Statistical Analysis

We estimated that, to assess correlations of at least *r* = 0.6 between CT and the other techniques assessed, a minimum sample size of 47 subjects was required to achieve a statistical power of 80% with a significance level of 5%.

Qualitative variables are expressed as absolute values and percentages. Differences between qualitative variables were analyzed using Chi-squared or Fisher tests. Quantitative variables are expressed as mean ± standard deviation. Normality was assessed using the Shapiro–Wilk test. Differences between quantitative variables were analyzed using Student’s *t*-test or Mann–Whitney U test, according to normality. Associations between variables were evaluated using Pearson’s or Spearman’s correlation coefficients, according to normality. Statistical significance for all analyses was set at *p* < 0.05 for bilateral contrast.

The diagnostic performance of CT parameters to detect malnutrition was assessed using receiver operating characteristic (ROC) curves and the area under the curve (AUC). Data analysis was performed using Jamovi version 2.3.2.

## 3. Results

A total of 55 patients were included. They had a mean age of 31.2 ± 9.3 years. A total of 33 were men (60%) and 22 were women (40%). A proportion of 43.6% of the individuals in our sample were heterozygous for ΔF508, and 69.1% had pancreatic insufficiency. They had a mean FEV1 of 66.8 ± 25.2% and a mean Bhalla score of 12.8 ± 5.1, without statistically significant differences in respiratory status between the Hospital Regional Universitario de Malaga and the Hospital Universitario Virgen del Rocío (FEV1 of 64.4 ± 28% vs. 69.1 ± 22.6%, *p* = 0.496; Bhalla score of 13.2 ± 5.5 vs. 12.4 ± 4.8, *p* = 0.584).

Only 5 (9.1%) patients had a BMI lower than 18.5 kg/m^2^. However, 19 (34.5%) presented low FFMI in BIA and 12 (21.8%) presented a BMI below 20 kg/m^2^, with a total of 24 (43.6%) patients diagnosed with malnutrition according to GLIM criteria.

Their general features are depicted in Table 1.

### 3.1. Correlation Between Computed Tomography and Other Body Composition Parameters

**Figure 3:**
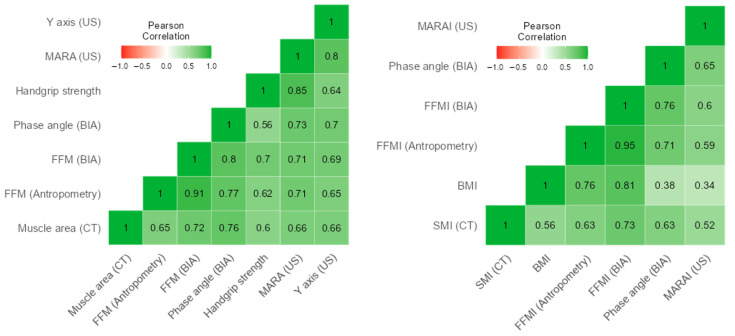
Correlation between Computed Tomography (CT) parameters and other body composition tools regarding muscle mass-related measurements.

Among the parameters associated with lean mass, muscle area assessed by CT was highly correlated with FFM measured by BIA (*r* = 0.725; *p* < 0.001), followed by anthropometry (*r* = 0.645; *p* < 0.001) and handgrip strength (*r* = 0.593; *p* < 0.001), whilst BMI presented the lowest correlation (*r* = 0.466; *p* < 0.001). Muscle area in CT also showed a high correlation with raw phase angle (*r* = 0.741; *p* < 0.001). SMI exhibited strong correlations with FFMI measured by BIA (*r* = 0.746; *p* < 0.001) and by anthropometry (*r* = 0.654; *p* < 0.001), and with raw phase angle (*r* = 0.648; *p* < 0.001), and a lower correlation with BMI (*r* = 0.572; *p* = 0.001). Additionally, 28 patients underwent muscle ultrasonography of the rectus femoris muscle. Muscle area in CT correlated with MARA (*r* = 0.657; *p* < 0.001) and the *Y*-axis (*r* = 0.650; *p* < 0.001). SMI also correlated with MARA (*r* = 0.523; *p* = 0.005) and the *Y*-axis (*r* = 0.563; *p* = 0.002) (Figure 3).

Regarding parameters related to fat mass, VAT, SAT, and IMAT areas correlated with fat mass measured by BIA (*r* = 0.716, *r* = 0.616, and *r* = 0.623, respectively; *p* < 0.001) and by anthropometry (*r* = 0.575, *r* = 0.562, and *r* = 0.574, respectively; *p* < 0.001), as well as with BMI (*r* = 0.634, *r* = 0.696, and *r* = 0.555, respectively; *p* < 0.001). No significant correlation was observed between SAT measured by CT and TSAT assessed by ultrasonography.

Finally, it is noteworthy that, although all patients diagnosed with exocrine pancreatic insufficiency in our cohort were receiving pancreatic enzyme replacement therapy, they exhibited a lower muscle area on CT compared to those without exocrine pancreatic insufficiency (95.7 ± 23.1 vs. 83.7 ± 21 cm^2^), showing a trend towards statistical significance (*p* = 0.063). No statistically significant differences were found between the two groups in terms of VAT (57.5 ± 49.4 vs. 45.2 ± 40.6 cm^2^, *p* = 0.335), SAT (65.6 ± 57.3 vs. 43.5 ± 41.0 cm^2^, *p* = 0.110), or IMAT (4.5 ± 4 vs. 3.3 ± 2.5 cm^2^, *p* = 0.250).

### 3.2. Respiratory Variables

Patients with no or mild pulmonary involvement according to the Bhalla score in the CT scan exhibited a lower prevalence of malnutrition according to GLIM criteria (27.6% vs. 61.5%, *p* = 0.011) and a better pulmonary function by spirometry (FEV1 78.5 ± 23.2% vs. 52.7 ± 20.1%, *p* <0.001), although no significant differences were observed in terms of exacerbations in the past year. These patients showed significantly higher values of muscle mass in the different tests performed including CT, as well as higher values of fat mass, although they did not reach statistical significance in all the tests performed (Table 2).

**Figure 4:**
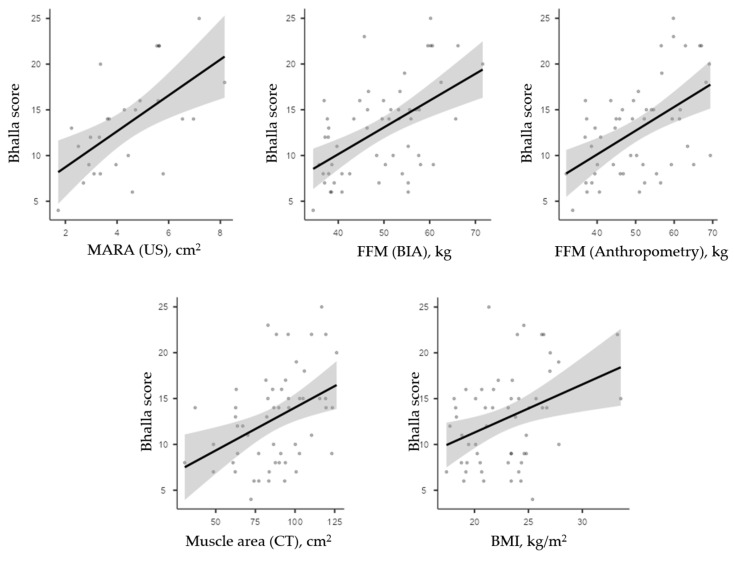
Correlation of Bhalla score with muscle mass-related parameters and BMI.

Additionally, we found positive statistically significant correlations between Bhalla score and muscle area in ultrasonography (*r* = 0.591; *p* = 0.001), FFM by BIA (*r* = 0.536; *p* < 0.001), FFM by anthropometry (*r* = 0.518; *p* < 0.001), and muscle area in CT (*r* = 0.403; *p* = 0.002). BMI alone showed a lower correlation with Bhalla score (*r* = 0.361; *p* = 0.007) (Figure 4).

We also found positive statistically significant correlations between FEV1 and Bhalla score (*r* = 0.717; *p* < 0.001), as well as between FEV1 and muscle area in CT (*r* = 0.488; *p* = 0.010).

### 3.3. Nutritional Status

A comparison between CT parameters according to nutritional status is shown in Table 3. Significant differences were found in muscle area and SMI between normo-nourished and malnourished PwCF.

**Figure 5:**
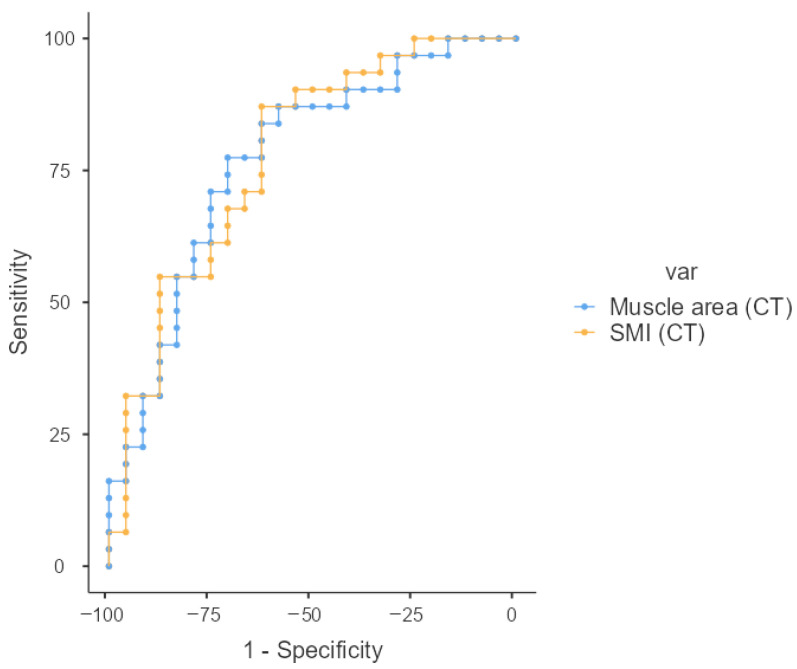
ROC curve analyses for muscle area and SMI to detect malnutrition. Sensitivity is represented on the *Y*-axis, with the inverse of specificity on the *X*-axis.

Using the ROC curve, we determined the muscle area and SMI cutoff points for predicting malnutrition (Figure 5). ROC curve analysis showed that both presented a significant discriminative ability to detect malnutrition. The muscle area cutoff for malnutrition diagnosis was 86.17 cm^2^ with AUC = 0.77 (sensitivity of 77.4% and specificity of 70.8%). The SMI cutoff for malnutrition diagnosis was 28.56 cm^2^/m^2^ with AUC = 0.78 (sensitivity of 87.1% and specificity of 62.5%).

## 4. Discussion

To our knowledge, there are few studies evaluating the usefulness of CT image analysis, used opportunistically, in PwCF. In our study, we observed that body composition parameters derived from T12 CT image analysis correlate well with anthropometry, BIA, handgrip strength, and ultrasonography in PwCF. In turn, we found an association between this tool and respiratory parameters and nutritional status.

Our sample comprises PwCF recruited from two hospitals, yet demonstrating homogeneous respiratory status across both centers. Their general characteristics closely resemble those reported in a larger multicenter Spanish study [21] as well as in the latest report of the Spanish Cystic Fibrosis Registry, recently published [22]. Additionally, they are comparable to other European cohorts, such as the adult subgroup of a Serbian investigation [7], whereas an Australian cohort demonstrated slightly poorer spirometric outcomes [23]. Furthermore, we observed that female participants in our sample exhibited a worse nutritional status compared to males. Consistent with these findings, Martínez et al. [24] previously reported that women with CF demonstrate significantly lower handgrip strength relative to healthy controls, while no such differences were observed among male patients. This suggests a potential sex-related disparity in muscle weakness within the CF population. Overall, up to 43.6% of our patients presented malnutrition according to GLIM criteria. Muscle mass depletion is associated with worse pulmonary function and greater disease severity in PwCF. Although BMI has traditionally been used to characterize nutritional status in this population, it does not accurately reflect body composition [25]. We previously noticed malnutrition underdiagnosis when using BMI compared to DXA [6]. King et al. [23] reported that using a BMI cutoff of 18.5 kg/m^2^ to identify malnutrition missed 58% of FFM depletion in PwCF, similarly to the study we present. As BMI alone does not accurately depict body composition in PwCF [25], nutritional assessment of this population should include body composition measurements beyond BMI [9].

However, no single method is considered the gold standard for body composition assessment in PwCF [25]. In this study, we used CT image analysis in an opportunistic way, since CT is considered a very useful tool for determining muscle mass quality and quantity, and for differentiation of adipose tissue subtypes [26].

Our study found good correlations between CT parameters and other body composition tools in PwCF. In a previous study in CF, Bryl et al. [27] manually drew paravertebral musculature in CT scans at the T12 level, and calculated muscle mass taking into account muscle volume and its average density, to compare with data obtained from clinic appointments of the same year, obtaining a CT-calculated muscle mass correlation with a BIA of *r* = 0.69, very similar to our results, and with a handgrip strength of *r* = 0.71, slightly higher than in our sample, in spite of using CT manual segmentation. In another study in PwCF in a pre-transplant situation, Jennerich et al. [28] found a correlation between SMI and BMI of 0.61, similar to our sample, using automatic segmentation with Slice-O-Matic software. Fernández et al. [29] used the same software as in our study to compare T12 CT image analysis with other morphofunctional assessment tools in people with idiopathic pulmonary fibrosis, obtaining the highest correlation of muscle area in CT with BIA parameters (*r* = 0.76–0.79) followed by muscle area in nutritional ultrasound (*r* = 0.62), similar to our results, but a lower correlation with handgrip strength (*r* = 0.46) than that found in our sample of PwCF, maybe because their population was predominantly elderly.

In our study, a notable association was observed between CT image analysis and respiratory parameters. Specifically, patients with a worse Bhalla score seemed to have lower values of lean mass-related parameters in all the techniques performed, including CT analysis. While causal relationships cannot be established, these findings align with existing evidence highlighting skeletal muscle mass as a valuable prognostic indicator in chronic respiratory diseases, including CF [27].

FEV1 correlated with Bhalla score in our study, as previously reported [21], and, to a lesser extent, also with muscle area on CT (*r* = 0.48). Bryl et al. [27] obtained a very similar correlation in their CF sample. We did not find a correlation between Bhalla score and exacerbations, in contrast to previous studies [21], possibly due to our smaller sample size and/or the situation of clinical stability of our patients, who presented a low number of total and severe exacerbations during the past year, probably influenced by treatment with ETI that some of them had already started.

Finally, we also found an association between CT image analysis and nutritional status: malnourished patients presented a significantly lower muscle area and SMI in CT. The results of our ROC curve for predicting malnutrition using these T12 CT parameters align with those previously obtained at the same vertebral level in people with idiopathic pulmonary fibrosis (muscle area of 80.5 cm^2^ and SMI of 28.8 cm^2^/m^2^) [29] and rectal cancer (SMI of 28.01 cm^2^/m^2^) [30].

Artificial intelligence-assisted CT image analysis is an emerging technique. L3 is currently considered the reference for CT-based body composition assessment [31], and several studies have already shown good correlations between CT at the L3 level and other body composition assessment techniques in pathologies such as obesity, colorectal cancer, or lung cancer [32,33,34]. However, PwCF often has chest CT as part of their routine clinical care [27]. Our results also indicate strong correlations between T12 CT-derived muscle measurements and other morphofunctional parameters, highlighting the consistency and reliability of using T12 for assessing muscle mass through CT in PwCF. Navas et al. [13] also suggested that T12 CT image analysis is a valid tool to assess body composition in PwCF. Additionally, in a previous study, Brath et al. [35] assessed thoracic muscle mass in Caucasian people without chronic disease, reporting a correlation of muscle area in T12 vs. L3 of *r* = 0.953, and a correlation of SMI in T12 vs. L3 of *r* = 0.931, similar to the rest of thoracic vertebra, concluding that any thoracic level can be used to assess muscle mass. Molwitz et al. [36] also found a strong correlation in muscle area between T12 and L3 (*r* = 0.80) in COVID patients, reinforcing the usefulness of this technique.

Based on our experience, since FocusedON-BC is a software tool that enables the extraction of nutritional information from an imaging test originally requested for a different purpose, analyzing these images using this software may serve as a useful screening approach to detect malnutrition without the need for the patient to be present. However, although FocusedON-BC has proven its effectiveness and reliability in several clinical studies, further research is required to validate this new tool.

This study had some limitations. Firstly, our sample size was relatively modest. As our study took place under routine clinical practice, the dates of CT scans did not coincide with those of the nutritional assessment. However, the estimated sample size was sufficiently met. Additionally, nutritional ultrasound was performed on a smaller sample of patients. However, the results are consistent with other measurements. Furthermore, the time elapsed between the CT scan and the assessment was up to 6 months, which may lead to minor changes during that period. As in any observational study, results should be interpreted within the appropriate population context, and causality can be suggested but not established. Finally, PwCF without CT scans performed during the established period were excluded, potentially leading to selection bias.

Nonetheless, as strengths of our study, we highlight that it includes an innovative artificial intelligence-assisted assessment of body composition, aimed at reducing intra- and interindividual variability. Additionally, several body composition techniques were used, which enhances the reliability of the results and brings it closer to clinical practice. Furthermore, this study was conducted in two centers using the same methodology, which facilitates generalization of the results. The exclusion of patients with treatment modifications, especially initiation of ETI, between our assessment and the CT scan, also reduced the variability associated with the clinical and nutritional improvement observed with these treatments. Finally, as an opportunistic test, the proposed technique does not require additional patient evaluations.

## 5. Conclusions

In conclusion, in adult PwCF, the assessment of body composition by CT image analysis correlates adequately with other body composition techniques such as anthropometry, BIA, handgrip strength, and nutritional ultrasound. Muscle area derived from CT analysis is related to the respiratory function and nutritional status of PwCF. However, further studies are required to validate this new tool and provide information on reference values, which could open a new avenue in the nutritional assessment of PwCF.

## Figures and Tables

**Table 1:** General features in the study sample, adjusted by nutritional status.

		Overall (*n* = 55)	Normo-Nourished (*n* = 31)	Malnourished (*n* = 24)	*p* Value ^a^
Age (years)	*m* ± SD	31.2 ± 9.3	32.7 ± 10.1	29.3 ± 8.0	0.195
Gender	*n* (%)				0.059
Men		33 (60)	22 (71)	11 (45.8)	
Women		22 (40)	9 (29)	13 (54.2)	
Mutation	*n* (%)				0.100
Homozygous for Δ-F508		15 (27.3)	5 (16.1)	10 (41.7)	
Heterozygous for Δ-F508		24 (43.6)	15 (48.4)	9 (37.5)	
Negative for Δ-F508		16 (29.1)	11 (35.5)	5 (20.8)	
Cystic fibrosis-related diabetes	*n* (%)	25 (45.5)	14 (45.2)	11 (45.8)	0.960
Pancreatic insufficiency	*n* (%)	38 (69.1)	18 (58.1)	20 (83.3)	0.044
Osteoporosis	*n* (%)	12 (21.8)	6 (19.4)	6 (25)	0.615
Total exacerbations	*m* ± SD	0.98 ± 1.07	1.07 ± 1.14	0.88 ± 0.99	0.519
Severe exacerbations	*m* ± SD	0.11 ± 0.37	0.07 ± 0.36	0.17 ± 0.38	0.331
FEV1 (%)	*m* ± SD	66.8 ± 25.2	74.9 ± 24.0	57.0 ± 23.6	0.009
FVC (%)	*m* ± SD	80.3 ± 20.0	84.9 ±19.5	74.5 ±19.3	0.062
FEV1/FVC (%)	*m* ± SD	0.67 ± 0.13	0.71 ± 0.11	0.63 ± 0.14	0.018
Colonizations	*n* (%)	50 (90.9)	29 (93.5)	21 (87.5)	0.643
*Pseudomonas aeruginosa*	*n* (%)	31 (56.4)	17 (54.8)	14 (58.3)	0.796
*Staphylococcus aureus*	*n* (%)	41 (74.5)	25 (80.6)	16 (66.7)	0.238
*Haemophilus influenzae*	*n* (%)	17 (30.9)	10 (32.3)	7 (29.2)	0.806
Bhalla Score	*m* ± SD	12.8 ± 5.1	14.5 ± 5.7	10.7 ± 3.4	0.004
Severity according to Bhalla Score	*n* (%)				0.011
Normal or Mild		29 (52.7)	21 (67.7)	8 (33.3)	
Moderate or Severe		26 (47.3)	10 (32.3)	16 (66.7)	
Treatment with ETI	*n* (%)	11 (20)	7 (22.6)	4 (16.7)	0.738

^a^ *p* values are derived from comparisons between normo-nourished and malnourished people. Abbreviations: ETI: Elexacaftor/Tezacaftor/Ivacaftor. FEV1: forced expiratory volume in 1 s; FVC: forced vital capacity; *m*: mean; SD: standard deviation.

**Table 2:** Differences between nutritional status, respiratory, and body composition parameters according to Bhalla score severity.

Bhalla Score Severity		Normal or Mild (*n* = 29)	Moderate or Severe (*n* = 26)	*p* Value
Malnutrition (GLIM)	*n* (%)	8 (27.6)	16 (61.5)	0.011
FEV1 (%)	*m* ± SD	78.5 ± 23.2	52.7 ± 20.1	<0.001
FVC (%)	*m* ± SD	88.2 ± 16.6	70.3 ± 19.6	0.001
FEV1/FVC (%)	*m* ± SD	0.72 ± 0.12	0.62 ± 0.11	0.005
Total exacerbations	*m* ± SD	1.1 ± 1.2	0.9 ± 0.9	0.524
Severe exacerbations	*m* ± SD	0.1 ± 0.4	0.1 ± 0.3	0.872
Anthropometry				
BMI (kg/m^2^)	*m* ± SD	23.9 ± 3.9	21.8 ± 2.8	0.023
FFM (kg)	*m* ± SD	53.8 ± 9.4	47.0 ± 10.2	0.017
FFMI (kg/m^2^)	*m* ± SD	18.3 ± 2.4	17.1 ± 2.1	0.056
FM (kg)		15.7 ± 7.9	12.8 ± 5.1	0.117
BIA				
Raw phase angle (º)	*m* ± SD	6.4 ± 0.8	5.9 ± 1.0	0.037
FFM (kg)	*m* ± SD	52.9 ± 8.7	45.0 ± 8.5	0.001
FFMI (kg/m^2^)	*m* ± SD	18.0 ± 2.3	16.5 ± 1.7	0.007
FM (kg)	*m* ± SD	16.7 ± 7.1	14.5 ± 7.4	0.283
Dynamometer				
Handgrip strength (kg)	*m* ± SD	35.3 ± 10.0	27.7 ± 7.6	0.003
CT				
Muscle area (cm^2^)	*m* ± SD	94.6 ± 21.1	79.3 ± 20.9	0.009
Muscle percentage	*m* ± SD	16.5 ± 2.7	15.9 ± 2.3	0.439
Muscle HU	*m* ± SD	39.0 ± 9.8	43.2 ± 9.4	0.110
SMI (cm^2^/m^2^)	*m* ± SD	32.2 ± 6.7	29.1 ± 6.5	0.083
IMAT area (cm^2^)	*m* ± SD	4.4 ± 3.8	2.8 ± 1.8	0.049
IMAT percentage	*m* ± SD	0.7 ± 0.5	0.5 ± 0.3	0.155
IMAT HU	*m* ± SD	−61.1 ± 5.7	−58.0 ± 5.1	0.038
VAT area (cm^2^)	*m* ± SD	58.9 ± 50.9	37.9 ± 30.5	0.067
VAT percentage	*m* ± SD	9.2 ± 6.5	7.2 ± 4.4	0.168
VAT HU	*m* ± SD	−90.1 ± 11.2	−86.6 ± 13.5	0.304
SAT area (cm^2^)	*m* ± SD	63.24 ± 55.7	35.9 ± 30.6	0.031
SAT percentage	*m* ± SD	9.9 ± 6.5	6.8 ± 5.1	0.056
SAT HU	*m* ± SD	−88.4 ± 15.1	−81.1 ± 17.1	0.099
Ultrasonography		**Normal or Mild (*n* = 15)**	**Moderate or Severe (*n* = 13)**	***p* Value**
MARA (cm^2^)	*m* ± SD	5.2 ± 1.6	3.4 ± 1.1	0.003
MARAI (cm^2^/m^2^)	*m* ± SD	1.8 ± 0.5	1.3 ± 0.4	0.010
*X*-axis (cm)	*m* ± SD	3.9 ± 0.6	3.5 ± 0.7	0.130
*Y*-axis (cm)	*m* ± SD	1.6 ± 0.3	1.1 ± 0.2	<0.001
TSAT (cm)	*m* ± SD	0.8 ± 0.4	0.9 ± 0.4	0.652

Abbreviations: BIA: bioelectrical impedance analysis; BMI: body mass index; FEV1: forced expiratory volume in 1 s; FM: fat mass; FFM: fat-free mass; FFMI: fat-free mass index; FVC: forced vital capacity; HU: Hounsfield unit; *m*: mean; IMAT: intramuscular adipose tissue; MARA: muscular area of rectus anterior; MARAI: muscular area of rectus anterior index; SAT: subcutaneous adipose tissue; SD: standard deviation; SMI: skeletal muscle index; TSAT: transverse subcutaneous adipose tissue; VAT: visceral adipose tissue.

**Table 3:** Differences between CT parameters according to nutritional status.

		Normo- Nourished (*n* = 31)	Malnourished (*n* = 24)	*p* Value
Muscle area (cm^2^)	*m* ± SD	96.3 ± 17.9	75.9 ± 22.1	<0.001
SMI (cm^2^/m^2^)	*m* ± SD	33.5 ± 4.9	27.1 ± 7.1	<0.001
IMAT area (cm^2^)	*m* ± SD	4.3 ± 3.3	2.8 ± 2.5	0.008
VAT area (cm^2^)	*m* ± SD	59.9 ± 48.2	34.9 ± 32.2	0.033
SAT area (cm^2^)	*m* ± SD	65.1 ± 53.2	31.2 ± 29.5	0.007

Abbreviations: IMAT: intramuscular adipose tissue; *m*: mean; SAT: subcutaneous adipose tissue; SD: standard deviation; SMI: skeletal muscle index; VAT: visceral adipose tissue.

## Data Availability

The original contributions presented in the study are included in the article, and further inquiries can be directed at the corresponding author.
